# A Single Eu-Doped In_2_O_3_ Nanobelt Device for Selective H_2_S Detection

**DOI:** 10.3390/s151229775

**Published:** 2015-11-30

**Authors:** Weiwu Chen, Yingkai Liu, Zhaojun Qin, Yuemei Wu, Shuanghui Li, Peng Ai

**Affiliations:** Institute of Physics and Electronic Information Technology, Yunnan Normal University, Kunming 650500, China; chenweiwu55@163.com (W.C.); qinzhaojun031@163.com (Z.Q.); wuyuemei893@163.com (Y.W.); lishuanghui808@163.com (S.L.); aipeng114@163.com (P.A.)

**Keywords:** Eu-doped In_2_O_3_, single nanobelt, gas sensor, H_2_S

## Abstract

Eu-doped In_2_O_3_ nanobelts (Eu-In_2_O_3_ NBs) and pure In_2_O_3_ nanobelts (In_2_O_3_ NBs) are synthesized by the carbon thermal reduction method. Single nanobelt sensors are fabricated via an ion beam deposition system with a mesh-grid mask. The gas-sensing response properties of the Eu-In_2_O_3_ NB device and its undoped counterpart are investigated with several kinds of gases (including H_2_S, CO, NO_2_, HCHO, and C_2_H_5_OH) at different concentrations and different temperatures. It is found that the response of the Eu-In_2_O_3_ NB device to 100 ppm of H_2_S is the best among these gases and the sensitivity reaches 5.74, which is five times that of pure In_2_O_3_ NB at 260 °C. We also found that the former has an excellent sensitive response and great selectivity to H_2_S compared to the latter. Besides, there is a linear relationship between the response and H_2_S concentration when its concentration changes from 5 to 100 ppm and from 100 to 1000 ppm. The response/recovery time is quite short and remains stable with an increase of H_2_S concentration. These results mean that the doping of Eu can improve the gas-sensing performance of In_2_O_3_ NB effectually.

## 1. Introduction

Due to its unique properties and special application prospects, low-dimensional metal-oxide semiconductor nanomaterials have been widely investigated in recent years [1,2,3]. For instance, Lu *et al.* have reported that Zn_2_GeO_4_ nanowires are prepared as a photoanode for quantum dot-sensitized solar cells and show an excellent performance [4]. For detecting poisonous or flammable gases, it is necessary to develop one-dimensional nano-scale gas sensors with high selectivity and sensitivity due to their fast response, low power consumption, and long-term reliability [5,6]. Among various active sensing materials, In_2_O_3_, as an n-type semiconductor with a wide band gap (~2.9 eV) and good chemical and thermal stabilities under practical operating conditions, has been widely used as a gas sensor [7,8]. Sun *et al.* have reported that In_2_O_3_ with appropriate mesostructured ordering has the potential to detect ethanol [9]. Lai *et al.* have reported that In_2_O_3_ nanorods have a good response to formaldehyde [10]. These investigations confirm that indium oxide nanomaterials really have good gas-sensitive properties. In order to gain higher response and selectivity, an additional noble metal as catalyst is efficient [11]. The effects of the addition of Au, Ag, La, and Ta on sensitive properties have been reported [12,13,14,15]. Shen *et al.* have found that Eu^3+^ can improve the performance of the bio-MOF-1 hybrid system for sensing organic amine vapors [16]. Hao *et al.* have reported that Eu can enhance sensing and electronic conductivity of metal-organic frameworks [17]. However, to the best of our knowledge, attention has been focused on the morphological or optical properties of Eu-doped In_2_O_3_ nanomaterials instead of their gas-sensitive properties [18,19]. Meanwhile, several features such as flexible structure, structural homogeneity, and crystallographic perfection make nanobelts a great choice for sensor devices [7]. Li *et al.* have obtained the formaldehyde gas-sensing properties of In_2_O_3_ nanofibers and nanobelts [20]. Their results showed that the highest response of the In_2_O_3_ nanobelt sensor (R_a_/R_g_ = 4.214 at 300 °C, where R_a_ is the sensor resistance in the air and R_g_ is the resistance in the tested gas) is higher than that of the In_2_O_3_ nanofiber sensor (R_a_/R_g_ = 3.113 at 340 °C). In addition, Ma *et al.* have found that the best working temperature of the SnO_2_ nanobelt sensor is 230 °C, which is much lower than that of tin dioxide nanoflower and porous nanosphere sensors (400 °C) reported by Hoa *et al.* [21,22]. The above-mentioned literature revealed that, compared to other nanodevices, nanobelt sensors have unique advantages. Therefore, there is a great demand to study the gas-sensing properties of Eu-In_2_O_3_ NBs.

In this paper, we present the synthesis of In_2_O_3_ NBs and Eu-In_2_O_3_ NBs by the carbon thermal reduction method. Then, the sensing properties of a single NB to five kinds of gases are measured. Compared with those of the pure NB, the Eu-In_2_O_3_ NB sensor has higher response and better selectivity to H_2_S. The Eu-In_2_O_3_ NBs show great potential in gas-sensing applications.

## 2. Experimental Section

The NBs were synthesized by the carbon thermal reduction method in a horizontal alundum tube (outer diameter of 4.0 cm, length of 100 cm) which was mounted inside a high-temperature tube furnace (HTF). For synthesizing Eu-In_2_O_3_ NBs, the mixture consists of In_2_O_3_, Eu (O_2_CCH_3_)_3_, and carbon powders (mass ratio~20:1:10). As for pure In_2_O_3_ NBs, the mixture is composed of In_2_O_3_ and carbon powders (mass ratio~2:1). The mixtures were put into a ceramic boat and then the boat was placed at the center of the HTF. A silicon wafer coated with 10-nm-thick Au film was put into the HTF with a distance of ~20 cm from the ceramic boat in the downstream and the tube was cleaned three times by argon gas. Then, the tube was evacuated by a mechanical pump to a pressure of 1–5 Pa. The center temperature of the HTF was increased to 1060 °C at a ramp rate of 10 °C/min and was maintained at this temperature for 120 min. In these processes, argon as a carrier gas was flowed at 20 sccm and oxygen was aerated at 10 sccm when the temperature of the HTF reached 600 °C. During the deposition process we adjusted the suction speed of the mechanical pump to keep the inside pressure 200 Torr. After the HTF was naturally cooled to room temperature, white materials deposited on the substrate were obtained.

The morphology, structure, and composition of the samples were characterized by X-ray diffraction (XRD), scanning electron microscopy (SEM), energy dispersive X-ray spectroscopy (EDS), transmission electron microscopy (TEM), and high resolution transmission electron microscopy (HRTEM).

**Figure 1:**
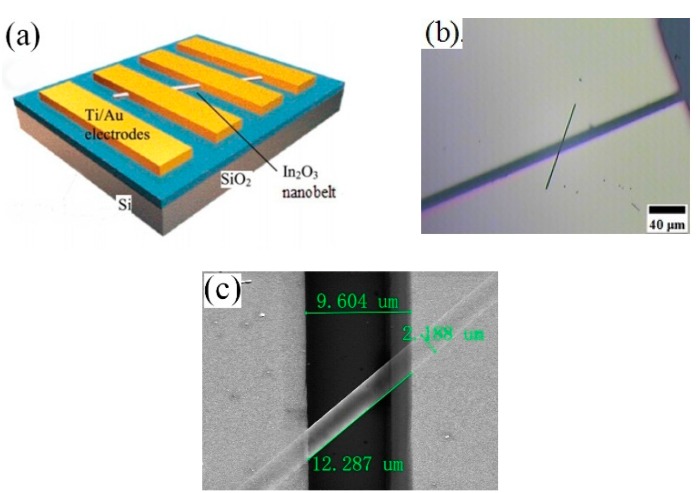
(**a**) A schematic diagram of the device; (**b**) The photo of the single nanobelt device; (**c**) SEM image of a single nanobelt device with Ti/Au electrodes on top of it.

In order to analyze the sensing performance of a single NB, the fabrication of the single nanobelt device was necessary. The NBs were first scratched by the tweezers and scattered in ethanol. Several drops of the soliquoid were dropped onto a p-type silicon substrate with a 500 nm thick SiO_2_ layer. After the ethanol evaporated completely, the Si substrate with the desired NB density was put into a mesh-grid mask and was deposited with inerratic Ti (8 nm) and Au (80 nm) electrodes by ion beam deposition. The background vacuum of the device was 1.0 × 10^−4^ Pa and the vacuum was held at 2.2 × 10^−2^ Pa during the deposition. In the deposition process, the argon was flowed and the flux was 10 mA/cm^2^. The schematic diagram of the prepared device is shown in Figure 1a and the optical microscopic image of a single nanobelt device is displayed in Figure 1b. Figure 1c presents the SEM image of Figure 1b, which is used for all gas-sensing measurements. The length and width of the measured nanobelt are about 12.287 μm and 2.188 μm, respectively. It is observed that two ends of the single nanobelt are covered with Ti/Au electrodes on top of it. The measurements were conducted in a hermetic stainless steel vessel (20 L) and the sensor was placed on a temperature control platform. The target liquid or gas would be injected into an evaporator to evaporate rapidly and the atmosphere in the chamber was made uniform by a fan. Finally, the gas-sensing performance of the prepared devices was measured by Keithley 4200.

## 3. Results and Discussion

### 3.1. Structures

**Figure 2:**
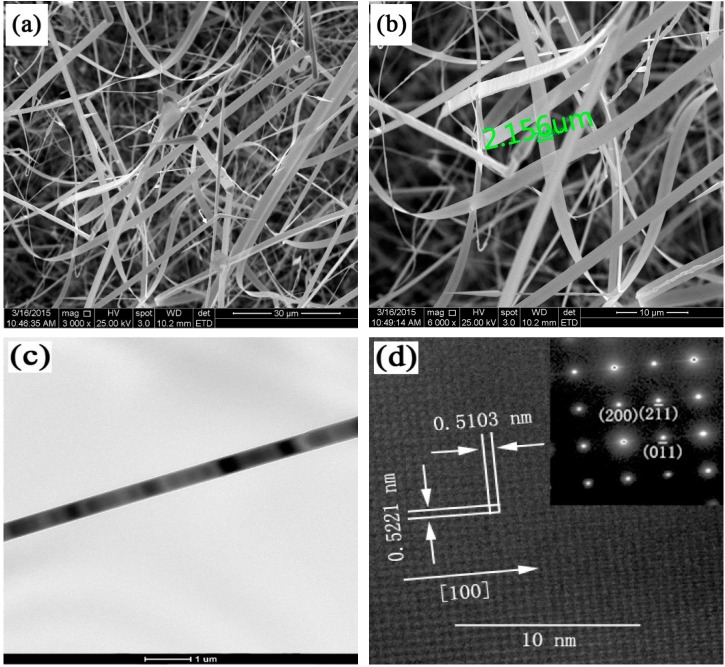
(**a**) Large-area SEM image; (**b**) Higher magnification SEM micrograph of Eu-In_2_O_3_ NBs; (**c**) TEM image of Eu-In_2_O_3_ NB; (**d**) HRTEM image, the inset: SAED pattern.

SEM images of Eu-In_2_O_3_ NBs are presented in Figure 2. It is seen that a large number of NBs were deposited on the substrate in Figure 2a. The length of the obtained NBs reaches several hundred micrometers. Figure 2b is the enlarged image of the local area of Figure 2a and shows that the width with uniform size is about several micrometers. Its surface is smooth and transparent, indicating that the thickness is very thin.

Figure 2c shows a TEM image of a Eu-In_2_O_3_ NB. It reveals that its width is about 480 nm. The HRTEM image and SAED pattern are displayed in Figure 2d and its inset. The interplanar spacings are 0.5221 nm and 0.5103 nm, corresponding to the (2 0 0) and (0 −1 1) crystal planes. The SAED pattern is indexed to the cubic In_2_O_3_ structure with a = b = c = 1.011 nm, indicating that it was a single crystal. Comparing HRTEM and SAED images, it is drawn that the growth direction of Eu-In_2_O_3_ NBs is along [1 0 1] and and no obvious structural defects exist.

**Figure 3:**
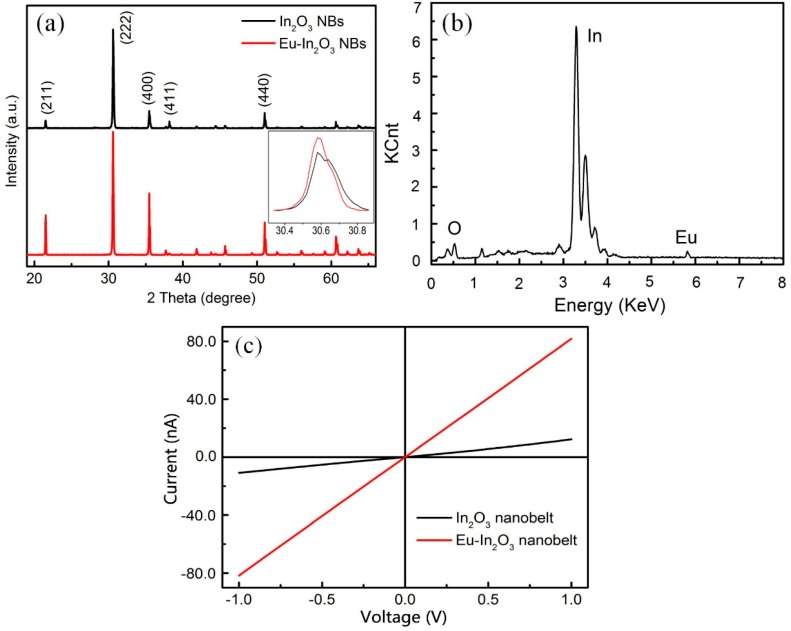
(**a**) XRD images of Eu-In_2_O_3_ and In_2_O_3_ NBs and the inset is the position of the peak at 30.6°; (**b**) EDS pattern of Eu-In_2_O_3_ NBs; (**c**) The I-V curves of the In_2_O_3_ NB and Eu-In_2_O_3_ NB.

The XRD patterns of Eu-In_2_O_3_ and In_2_O_3_ NBs are displayed in Figure 3a. It shows that all well-defined diffraction peaks can be indexed as the cubic In_2_O_3_ phase with a = b = c = 1.011 nm (JCPDS Card No.06-0416). The prepared sample is well crystallized and no diffraction peaks of other impurities or crystalline by-products are detected. The inset of Figure 3a illustrates that the peak (located at ~30°) of Eu-In_2_O_3_ shifts to a lower angle compared with that of In_2_O_3_ (at 30.6°), indicating that the constants of the latter are larger than those of the former because the radius of Eu ions (94.7 pm) is larger than that of In ions (80 pm). This result reveals that Eu is doped in the lattice. To further make sure whether Eu is doped into the lattices of In_2_O_3_ or not, an energy-dispersive X-ray spectroscopy (EDS) of Eu-In_2_O_3_ NBs was carried out, as shown in Figure 3b. It confirms that Eu has been doped into the nanobelts and the content of Eu is 0.86 wt.%. Figure 3c presents I-V curves of the In_2_O_3_ NB and Eu-In_2_O_3_ NB. It is noted that the curves are nearly linear, revealing that good ohmic contacts are formed between the Eu-In_2_O_3_ NB/In_2_O_3_ NB and the electrodes. Besides, the resistance of the Eu-In_2_O_3_ NB is lower than that of its pure counterpart, indicating that the dopant improves the conductance of the In_2_O_3_ NB.

### 3.2. Sensing Properties

**Figure 4:**
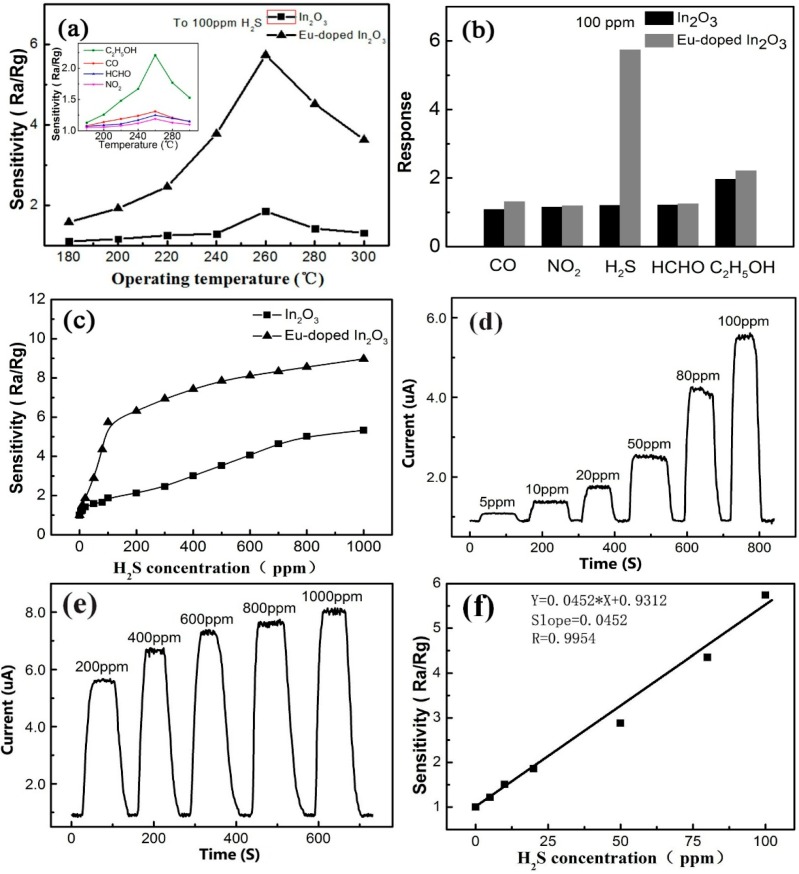
(**a**) The sensitivity curves of Eu-In_2_O_3_ NB and In_2_O_3_ NB to 100 ppm of H_2_S at different temperatures and the inset is the sensitivity curves of Eu-In_2_O_3_ NB to different gases at different temperatures; (**b**) Histogram of two devices responding to different gases at 260 °C; (**c**) Response curves of Eu-In_2_O_3_ NB to H_2_S at 5~1000 ppm at 260 °C; (**d**) Responses curve of Eu-In_2_O_3_ NB to H_2_S at 5~100 ppm at 260 °C; (**e**) Response curve of Eu-In_2_O_3_ NB to 200~1000 ppm of H_2_S at 260 °C; (**f**) Fitting the curve of response *versus* H_2_S concentration in the range of 5–100 ppm.

The sensitivity is defined as R_a_/R_g_ where R_a_ is the sensor resistance in the air and R_g_ is the resistance in the tested gas or R_g_/R_a_ at an oxidizing one. Figure 4a shows the sensitivity curves of the Eu-In_2_O_3_ NB and In_2_O_3_ NB to 100 ppm of H_2_S at different temperatures and its inset shows the sensitivity curves of the Eu-In_2_O_3_ NB to different gases at different temperatures. It is obvious that the optimum operating temperatures of the two devices to five tested gases are 260 °C. Furthermore, the response of the Eu-In_2_O_3_ device to H_2_S reaches 5.74, which is five times that of its pure counterpart. The histogram of two devices corresponding to different gases at 260 °C is shown in Figure 4b. The responses to 100 ppm of CO, NO_2_, HCHO, and C_2_H_5_OH at 260 °C are only 1.31, 1.25, 1.19, and 2.21, respectively. The response to H_2_S is several times higher than that of the other four gases, meaning this sensor is more sensitive to hydrogen sulfide. It is noted that the doping of Eu obviously improves the response to H_2_S. Although it also increases the responses to other gases, they are not outstanding compared to H_2_S.

The responses to different H_2_S concentrations at 260 °C are tested, as shown in Figure 4c. It is seen that there is a linear relationship between the response and H_2_S concentrations when the concentration changes from 0 to 100 ppm and from 100 to 1000 ppm. It is noted that the slope of 5 to 100 ppm is higher than that of 100 to 1000 ppm, illustrating that the response increases slowly in this range. The surface coverage tends to saturate and leads to the slope getting smaller at higher concentrations [23] in that the surface coverage of the adsorbed molecules follows the Langmuir isotherm. In our experiment, the minimum detection concentration of the Eu-In_2_O_3_ sensor is about 5 ppm to H_2_S. As reported in the literature, though the minimum detection concentration of In_2_O_3_ can reach about several hundred ppb to H_2_S, the specific surface area of these nanostructures is always much larger [8]. For instance, Zhao *et al.* have reported the lowest detection concentration limit of a In_2_O_3_ nanotube device could reach 500 ppb for H_2_S, but that device is composed of many nanotubes, not a single one [5].

Figure 4d shows the responses curves of Eu-In_2_O_3_ NB to 5~100 ppm of H_2_S at 260 °C and it is seen that six cycles are recorded, corresponding to 5, 10, 20, 50, 80, and 100 ppm of H_2_S. It can be seen that the resistance of In_2_O_3_ NB declines significantly upon injection of H_2_S and returns to its original state when H_2_S is expelled. Response/recovery time is an important parameter for a gas sensor. For 5~100 ppm of H_2_S, the response (recovery) time of the Eu-In_2_O_3_ NB is 9 (11), 11 (11), 13 (14), 10 (13), 12 (18), and 11 (13) s, respectively. Obviously, the response/recovery time changes little with an increase of H_2_S concentration and is less than 18 s, manifesting fast response speed. In addition, the response curves of the Eu-In_2_O_3_ NB to H_2_S at high concentrations at 260 °C are shown in Figure 4e. Repeated measurements have been carried out at each concentration and the results are stable and reliable, indicating that the nanobelt device possesses good repeatability and stability.

Fitting the curve of response *versus* H_2_S concentration in the range of 5–100 ppm is presented in Figure 4f. It can be seen that the slope of the curve is 0.0452 ppm^−1^ with a fitting quality of R = 0.9954. The sensor noise is calculated by the variation in the relative response in the baseline with help of root-mean-square deviation (RMSD) [24,25]. Then, 120 data points (N) of Figure 4d at the baseline are collected, and the standard deviation (S) is obtained as 0.0932. According to RMS_noise_ = $\sqrt{{\text{S}}^{2}/\text{N}}$, RMS_noise_ is 0.0085 for the H_2_S sensor. The theoretical detection limit of the sensor is 0.564 ppm based on the signal-to-noise ratio using DL (ppm) = 3 × (RMS_noise_/Slope).

### 3.3. Sensing Mechanism

In_2_O_3_ is an n-type semiconductor and free electrons are major charge carriers [26]. The In_2_O_3_ NB adsorbs oxygen-negative ions (O^2−^, and O^−^) resulting from a combination of oxygen molecules and electrons by Equations (1) and (2), which makes the electrical conductivity of In_2_O_3_ NB reduce [27,28].

O_2_ + e^−^ → O_2_^−^ (1)

O_2_^−^ + e^−^ → 2O^−^ (2)

The following reactions will occur on the surface of it when In_2_O_3_ NB is in the strong reducing atmosphere of H_2_S.

2H_2_S + 3O_2_^−^ = 2H_2_O + 2SO_2_ + 3e^−^ (3)

H_2_S + 3O^−^ = H_2_O + SO_2_ + 3e^−^ (4)

According to these reactions, the electrons are released, and the conductivity of the nanobelt is enhanced. On one hand, rare earth ions can promote these reactions as a catalyst, leading to the improvement of the sensitivity [29]. On the other hand, the doping of Eu can effectively increase the number of oxygen vacancies because In_2_O_3_ NBs belong to an n-type semiconductor and oxygen vacancies usually provide donor states [30,31]. Thus, the electric conductivity of In_2_O_3_ NBs enhances.

## 4. Conclusions

Eu-doped In_2_O_3_ NBs and pure In_2_O_3_ NBs have been prapared by the carbon thermal reduction method. The sensitive properties of two devices based on a single Eu-In_2_O_3_ NB and its undoped counterpart have been measured using various gases at different concentrations and temperatures. It is found that the response of the Eu-In_2_O_3_ device to 100 ppm H_2_S reaches 5.74, which is fivetimes that of its undoped counterpart, at 260 °C. A linear relationship between the response and different H_2_S concentrations is obtained when the concentration changes from 5 to 100 ppm and from 100 to 1000 ppm. The theoretical detection limit of the Eu-doped In_2_O_3_ NB sensor is 0.564 ppm at optimum working temperature. It reveals that the doping of Eu improves the sensing performance of In_2_O_3_ NB effectively and Eu-In_2_O_3_ NBs have the potential to be fabricated as H_2_S sensors.
